# Genome-Wide Identification and Characterization of *wALOG* Family Genes Involved in Branch Meristem Development of Branching Head Wheat

**DOI:** 10.3390/genes9100510

**Published:** 2018-10-19

**Authors:** Wenzhi Nan, Shandang Shi, Diddugodage Chamila Jeewani, Li Quan, Xue Shi, Zhonghua Wang

**Affiliations:** 1 State Key Laboratory of Crop Stress Biology for Arid Areas, College of Agronomy, Northwest A&F University, Yangling 712100, China; nanwenzhi1@163.com (W.N.); shishandang@nwsuaf.edu.cn (S.S.); jeewanididdugoda@yahoo.com (D.C.J.); lquan@nwafu.edu.cn (L.Q.); 2 College of Life Science, Yulin University, Yulin 719000, China; 3 Grain Legumes and Oil Crops Research and Development Centre, Department of Agriculture, Angunakolapelessa 82220, Sri Lanka

**Keywords:** branching head wheat, wheat *ALOG*, phylogenetics, gene expression, sequence diversification, cis-acting regulatory elements

## Abstract

The branched spike phenotype is an important supernumerary spikelet trait of *Triticum turgidum* L. associated with the production of significantly more grains per spike, thereby offering a higher potential yield. However, the genetic basis of branch meristem (BM) development remains to be fully elucidated in wheat. *TAW1*, an *ALOG* (Arabidopsis *LSH1* and Oryza *G1*) family gene, has been shown to function as a unique regulator in promoting BM development in rice. In this study, we found that the development pattern of the BMs of the branched spike in wheat was similar to the indeterminate BMs of rice. Moreover, phylogenetic analysis classified the *ALOG* genes into 12 groups. This family of genes was found to have evolved independently in eudicots and monocots and was evolutionarily conserved between wheat and rice as well as during wheat polyploidization. Furthermore, experiments revealed that *TtALOG2-1A*, a *TAW1*-homologous gene, plays a significant role in regulating the transition of indeterminate BM fate. Finally, large-scale RNA-sequencing studies and quantitative real-time polymerase chain reaction (qRT-PCR) experiments revealed that members of the *TtALOGs* may act upstream of the *TtMADS22*, *TtMADS47*, and *TtMADS55* genes to promote indeterminate BM activities. Our findings further knowledge on BM development in wheat.

## 1. Introduction

Cereal crops are the main source of carbohydrates for humans, and the yields of these crops are largely dependent on the inflorescence architecture [1,2,3], which is strongly dictated by the activity and determinacy of different types of meristems. For example, the inflorescence of rice involves shifts from the inflorescence meristem (IM) to the branch meristem (BM), followed by the spikelet meristem (SM) and then the floral meristem (FM) [4]. An indeterminate IM may initiate either BMs or SMs, and a BM ultimately converts into an SM. The transition from the IM to SM is crucial for lateral BM development [5,6]. Generally, delaying SM transition results in the formation of more BMs and leads to larger inflorescences. The genes that regulate the determinacy and fate transitions of IMs into SMs include *FZP* [7], *APO1* [8], *APO2* [9], and *TAW1* [5] in rice. Interestingly, *TAW1* encodes a nuclear protein with a conserved ALOG (Arabidopsis *LSH1* and Oryza *G1*) domain and it may function as a unique regulator in promoting IM activity, inhibiting the phase change to SM identity [5].

In general, the indeterminate IMs of wheat can only initiate conversion into SMs, followed by the initiation of glume primordia (GP), lemma primordia (LP), and FMs from SMs, but fail to initiate BMs [10,11]. However, in wheat with the branching head trait, branched spikes can be produced below the middle of the inflorescence with significantly more grains per spike and higher growth potential. The pattern of the branched spike provides an opportunity to increase our understanding of BM development in wheat. To date, the characterized molecular regulators for the determinacy and fate transitions of IMs into SMs are rudimentary in wheat, including *Ppd-1* and *WFZP* genes, but the gene regulating BM development remains unknown. *Ppd-1* acts as a key regulator of paired spikelet formation in wheat; it delays the conversion of IMs into SMs [12]. Furthermore, the *wfzp-A*/*bht-A1* or *wfzp-D* mutants of wheat cause additional spikelet formation but not spike branching due to lost SM determinacy [13,14].

Members of the *ALOG* gene family have been functionally characterized and shown to regulate the inflorescence architecture by mediating the phase transition of IMs due to their roles in the maintenance of undifferentiated cells, including *LSH1*, *G1*, *TH1/BSG1*, and *TAW1* [5,6,15,16,17,18]. The Arabidopsis *LSH1* and Oryza *G1* family proteins contain an ALOG domain, which has 10 members both in Arabidopsis and in rice. *LSH1* was the first identified *ALOG* gene in eudicots, the overexpression of which suppresses the phase transition of IMs into FMs and the transformation of flowers into inflorescence shoots [15]. *LSH3* and *LSH4* are involved in the suppression of organ differentiation in boundary regions, and the ectopic expression of these proteins results in the formation of extra flowers, extra floral organs, or chimeric floral organs within a flower. However, the detailed functions of *LSH3* and *LSH4* remain unclear [19]. In monocots, rice *G1* was the first identified *ALOG* gene and *g1* mutant plants show empty glumes that transform into lemma-like organs [16,20,21,22]. *TH1/BSG1* functions as a transcriptional repressor regulating lemma and palea development in rice [18,23,24,25]. Finally, *TAW1* has been proven both necessary and sufficient to regulate BM development in rice, and the increased expression of the TAW1 protein leads to enhanced indeterminate BM activity and delayed determinate SM fate [4,5].

ALOG family genes, corresponding to Domains of Unknown Function 640 (DUF640) proteins in the protein-family (Pfam) database, are among several gene families that encode functionally uncharacterized proteins. In eukaryotes, DUFX proteins now contain over 4885 DUF families, where X represents the order of addition to the Pfam database [26]. Different DUF families play various roles in stress responses and plant development [5,16,19,27,28]. Although the precise function of the ALOG domain remains uncertain, the characteristics of sequence-specific DNA binding and homodimer formation suggest a role as a specific transcription factor in plants [18,27]. The ALOG family of proteins is present in multiple copies in land plants. All members of this family show a highly conserved ALOG domain region, whereas the N- and C-termini are highly diverse in monocots and eudicots [18,29]. A nuclear localization signal, KKRK, was identified in the C-terminal flanking region after the ALOG domain.

It is well-established that allohexaploid wheat (*Triticum aestivum* L., AABBDD, 2n = 6× = 42) originated from three diploid ancestral species: an A-genome donor of *Triticum urartu* (AuAu, 2n = 2× = 14), an ambiguous B-genome donor of a related organism (SS, 2n = 2× = 14), and a D-genome donor of *Aegilops tauschii* (DD, 2n = 2× = 14). First, wild tetraploid emmer wheat *Triticum turgidum* ssp. Dicoccoides (AABB, 2n = 4× = 28) emerged from crosses of *T. urartu* and the B-genome donor. Then, domesticated emmer wheat *T. turgidum* was involved in hybridization with *Ae. tauschii*, followed by whole genome duplication, giving rise to hexaploid bread wheat [30,31]. The duplication of genomes is a major force of evolution that affects gene copy number [32,33]. There is significant evidence that massive duplicate gene silencing and elimination has frequently occurred throughout wheat evolution, and that the loss of duplicate genes is not a random process [34,35,36]. Recent advances in wheat genomics provide the opportunity to identify and characterize wheat *ALOG* (*wALOG*) family genes in hexaploid wheat and its ancestral genome during allopolyploidization. The availability of reference quality genome sequences for *T. urartu* [37], *Ae. tauschii* [38], *T. turgidum* ssp. Dicoccoides [39], and *T. aestivum* L. [31,40] has enabled more definitive analysis of the genetic evolution, cis-regulated elements of promoters, protein motifs, and expression patterns of multicopy gene families in wheat. Furthermore, since the *ALOG* gene family has previously been reported to function in regulating indeterminate BM fate, we used branching head wheat in this study to further investigate the role of *wALOG* genes in BM development.

Here, we systematically characterized *wALOG* families in wheat by bioinformatic analysis. Further, the global expression patterns of *TtALOG* genes and potential downstream effects of six *TtSVP* subfamily genes were analyzed by large-scale RNA-sequencing (RNA-seq) studies and quantitative real-time polymerase chain reaction (qRT-PCR) experiments in branching head wheat to investigate BM development. The *ALOG* genes, which may regulate indeterminate BM fate, and their promoter were also cloned by the Sanger Sequencing Centre. The analyses presented in this paper provide new insight into the role of the ALOG family of proteins in the wheat BM identity.

## 2. Materials and Methods

### 2.1. Plant Material and Growth Conditions

Tetraploid wheat (*Triticum turgidum* L., AABB, 2n = 4× = 28) GAN-A1582 (spikes of a normal head) and GAN-A631 (spikes of a branching head) were grown in the field at the experimental station in Yangling (34.16° N, 108.05° E) on October 4 of 2014, 2015, and 2016. Because environmental conditions can affect the formation of the branching head, samples from three consecutive years were used for this study. For each year, three replicates were arranged using the randomized complete block design, and four stages of tissues were collected from each replication: the early double ridge (EDR), late double ridge (LDR), BM development, and SM-initiated (SMI) stages in GAN-A631 and the EDR, LDR, GP, and LP stages in GAN-A1582. Samples of three replicates (10 spikes per replication) from each stage per year were mixed together. For each stage, three pooled spikes in 2014, 2015, and 2016 were used for three biological replicates in RNA-Seq and qRT-PCR studying. The tissues of the inflorescences from developmental stages were distinguished based on the characteristics of the meristem of each stage which were observed using a stereomicroscope (Leica S8 APO, Leica Microsystems, Wetzlar, Germany), and the sample of each stage were conserved by immediate freezing in a 2 mL microcentrifuge tube suspended in liquid nitrogen, followed by storage at −80 °C.

### 2.2. Scanning Electron Microscopy 

Scanning Electron Microscopy (SEM) was performed on immature spike tissues including the EDR, LDR, BM development, and SMI stages in GAN-A631 and the EDR, LDR, GP, and LP stages in GAN-A1582 from field-grown plants. SEM was conducted as described previously [41].

### 2.3. RNA Isolation and RNA Sequencing

The RNAs of three biological replicates of GAN-A631 were extracted using the Trizol reagent (TaKaRa, Otsu, Japan). RNA was quantified and integrity was assessed using an RNA 6000 Nano Assay bioanalyzer (Agilent, Santa Clara, CA, USA). In total, twelve sequencing libraries of four stages were constructed by standard Illumina library preparation methods using the NEB Next Ultra RNA Library Prep Kit (NEB, Ipswich, MA, USA). Paired-end sequencing libraries with an insert size of approximately 250 bps were sequenced on an Illumina HiSeq 2500 sequencer (Illumina, San Diego, CA, USA). The results were submitted into the NCBI SRA database (SRP159158).

### 2.4. RNA-seq Data Processing

For the raw reads, the adaptor sequences, the reads in which unknown bases represented more than 10% of the total bases, and the low-quality reads (percentage of low-quality bases with a quality value ≤ 20 in more than 50% of a read) were removed using Trimmomatic software (version 0.32) [42]. The sequencing data quality was verified using FastQC software (version 0.10.1) [43]. Filtered reads were mapped to the reference genome of wild tetraploid emmer wheat *T. turgidum* ssp. Dicoccoides (downloaded from WEWseq) with HISAT2 (version 2.1.0) [44]. The Cufflinks (version 2.2.1) [45] was used to reconstruct the transcripts. The uniquely mapped reads number of each gene model was determined using HTSeq (version 0.6.1) from BAM alignment files [46]. Then, read counts were normalized using the fragments per kilobase transcriptome per million mapped reads (FPKM) [47]. The *TtALOG*, *TtMADS22*, *TtMADS47*, and *TtMADS55* expression patterns in RNA-seq data were analyzed with the Complex Heatmap R package [48].

### 2.5. Isolation of ALOG Family Genes

The protein sequences of the ALOG family members of Arabidopsis and *Oryza sativa* were downloaded from Ensembl Plants [49]. The coding sequence (CDS), protein, and genome sequences of *T. urartu*, *Ae. tauschii*, wild tetraploid emmer wheat *T. turgidum* ssp. Dicoccoides, and *T. aestivum* were retrieved from the databases MBKBASE [50], the *Ae. tauschii* genome (http://aegilops.wheat.ucdavis.edu/ATGSP/), WEWseq [51] and Ensembl Plants [49], respectively. We followed two approaches to identify *ALOG* family genes in wheat. In the first approach, the local BLAST database of CDS sequences of each wheat species was established; then, the 20 previously identified ALOG protein sequences [15,52] presented in rice and Arabidopsis were used as queries and tblastn searches were performed against the local BLAST database. Tblastn analyses were performed using blast 2.2.28+ (ftp://ftp.ncbi.nih.gov/blast/executables/LATEST/) with the default parameters and an E-value cutoff of 1 × 10^−5^. In the second approach, the annotated wheat proteome was used as a query to search for the family-specific DUF640 domain (PF04852) hidden Markov model (HMM) profiles obtained from the Pfam database [53]. HMMER v3.0 was employed to perform an HMM search with the default parameters and an E-value cutoff of 1 × 10^−5^ [54]. These two results were merged to remove redundancy in MEGA6 [55] and examined for the presence of the conserved domain in the conserved domain database of NCBI [56].

### 2.6. Multiple Sequence Alignment, Motif Identification, and Phylogenetic Analysis

Multiple sequence alignments for the ALOG family proteins were performed using Muscle v3.8.31 under the default settings [57]. Conserved motifs of ALOG amino acid sequences were analyzed using the MEME website [58] with the following parameters, max number of motifs, 10 and min/max motif width, 6–50 (inclusive). A neighbor-joining tree of these aligned proteins was created using RAxML v8.2.10 [59] with the default parameters and 1000 bootstrap replications. The resultant phylogenetic tree was edited and displayed using the ITOL v4 program [60].

### 2.7. Cis-Acting Regulatory Elements Prediction in the Promoter Regions of *wALOG* Genes

The promoter regions (2000 bp of the upstream sequence) of the *ALOG* genes were extracted from the draft genome of wheat (*T. aestivum* and *T. turgidum* ssp. Dicoccoides) using the bioinformatic tool Tbtools v0.52 [61]. The conserved cis-acting regulatory elements present in the promoter regions were predicted using the PlantCARE database [62].

### 2.8. qRT-PCR Analysis

RNAs were isolated from tissues of the four developmental stages (EDR, LDR, BM development, and SMI stages in GAN-A631; EDR, LDR, GP, and LP stages in GAN-A1582). cDNA synthesis was performed with a PrimeScript^®^ RT Reagent Kit (Perfect Real Time; TaKaRa Bio, Shiga, Japan) according to the manufacturer’s protocol. Three independent biological replicates for each stage were obtained over three years and three technical replicates of each biological replicate were arranged for real-time PCR analysis with a 7500 Fast Real-Time PCR System (Applied Biosystems, Foster City, CA, USA). Each reaction was prepared using 10 μL of 2× SYBR Premix Ex Taq (TaKaRa), 2 μL of 20 ng/μL cDNA, and 0.4 μL of 10 μM forward and reverse primers, in a total volume of 20 μL. The PCR conditions were 30 s at 95 °C, followed by 40 cycles of 95 °C for 5 s, and Tm ± 2 °C for 20 s. The relative expression levels of candidate genes were represented in the form of relative fold changes using the following formula: FC = 2 −Δ(ΔCt) [63]. Quantitative RT-PCR primer sequences are listed in Table S1.

### 2.9. Cloning of BM Development-Associated TtALOG Genes and its Promoters

Gene-specific primers or promoter-specific primers were designed on the basis of transcripts reconstructed from RNA-seq data and the genomes of *T. aestivum* and *T. turgidum* ssp. Dicoccoides. Primers were designed using Premier 5.0 software [64]. *T. turgidum* L. cDNA libraries, which were used for the cloning of *TtALOG* genes, were reverse-transcribed from the mixed RNAs of the four stages of tissues using the PrimeScript^®^ RT reagent kit (TaKaRa, Beijing, China) according to the manufacturer’s instructions. Genomic DNA was extracted from the leaves of *T. turgidum* L. to obtain the promoter region using the CTAB method [65]. The PCR products of *TtALOG* genes and their promoters were further purified and ligated into pMD 18-T vector (TaKaRa). *E. coli* DH5α and the pMD 18-T vector were used for gene cloning and sequencing, respectively. The primers are represented in Table S1.

## 3. Results

### 3.1. Morphological Differences and Lateral BM Initiation of Branching Head Wheat

**Figure 1:**
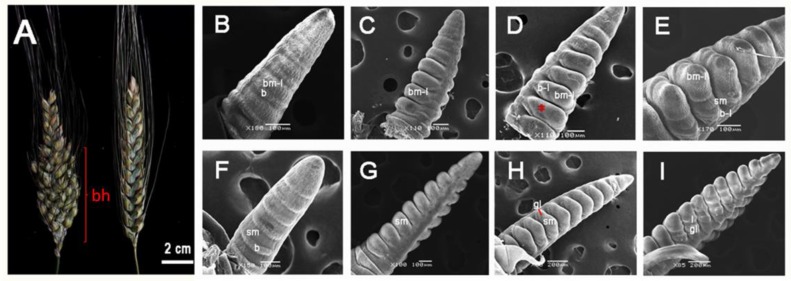
Morphology of the spikes of a normal head (nh) and the spikes of a branching head (bh). (**A**) Spike morphology of GAN-A631–left side (bh) and GAN-A1582–right side (nh). (**B**) The early double ridge (EDR) stage of bh. (**C**) The late double ridge (LDR) stage of bh. (**D**) The branch meristem (BM) development stage of bh and the red asterisk indicates the region of secondary spikelet meristem (SM) emergence. Compared with the glume primordia (GP) stage of nh, slight bulging can be observed at the location of the red asterisk in bh. (**E**) The SM initiation (SMI) stage of bh. (**F**) The EDR stage of nh. (**G**) The LDR stage of nh. (**H**) The GP stage of nh. (**I**) The lemma primordia (LP) stage of nh. Abbreviations: bm-l, branched-like meristem; b, bract; b-l, bract-like meristem; sm, spikelet meristem; gl, glume; l, lemma. Scale bars: (B–G), 100 µm; (H,I), 200 µm.

The early stages of inflorescence development in wheat with a normal head have been described in previous studies [10,11]. Here, the morphological differences in inflorescence development between GAN-A1582 (spikes of a normal head) and GAN-A631 (spikes of a branching head) were analyzed using SEM. In GAN-A1582, the IMs did not produce any lateral BMs from the inflorescence axis, and the SMs were alternately arranged as two opposite rows on the main axis (Figure 1A,F,G). However, in GAN-A631, long lateral branches formed in the middle and lower parts of the main axis (Figure 1A), and the SMs reverted to BMs from which secondary SMs initiated (Figure 1D,E).

Based on morphological changes, the early stages of inflorescence development in GAN-A1582 can be classified into four stages: EDR, LDR, GP, and LP. When SMs visibly emerged together with the bract primordia, they appeared phenotypically double-ridged in the EDR stage (Figure 1F). The obvious swelling of the SMs was associated with the degeneration of the bract primordia, whereas the bract primordia almost disappeared in the LDR stage (Figure 1G). Each SM initiated a glume primordium in the GP stage (Figure 1H), after which the LP appeared in the LP stage (Figure 1I). However, the EDR, LDR, BM development, and SMI stages were classified in GAN-A631. In GAN-A631, the early inflorescence morphological changes coincided with the EDR and LDR stages of GAN-A1582, and we designated these stages as EDR (Figure 1B) and LDR (Figure 1C), respectively. IMs acquired a lateral BM identity in the EDR stage of GAN-A631, and then the lateral BMs swelled in the LDR stage. The emergence of bract-like meristems was observed in the basal portion of lateral BMs in the BM development stage (Figure 1D), and then SMs formed in the SMI stage (Figure 1E).

### 3.2. RNA-Seq Analysis of Early Stages of Inflorescence Development in GAN-A631

In total, 12 sample libraries were constructed and sequenced. We obtained 44.22 to 72.89 million reads from each sample (Table 1). After cleaning and checking the read quality, 42.33 to 70.73 million clean reads were generated, where the clean data GC content ranged from 52.31% to 55.94% in different libraries, and the Q20 percentage exceeded 94.24%. These libraries contained 87.34 to 93.10% of mapped clean reads to the reference genome of wild tetraploid emmer wheat *T. turgidum* ssp. Dicoccoides. Based on the clean reads, 47,809 expressed genes (FPKM > 1 in one or more than one of the twelve sample libraries) were detected, including 44,699 known genes and 3110 novel genes. We prepared RNA samples from the spikes in the EDR to SMI stages of GAN-A631, and thus our gene expression data mainly reflected the formation of branch-like meristems to SMs in branching head wheat.

### 3.3. Expansion of the wALOG Family Genes During Wheat Polyploidization

To characterize the copy number variation of *wALOGs* during wheat polyploidization, we identified the ALOG family members in hexaploid wheat and its relatives (diploid and tetraploid wheat). In total, the *T. urartu*, *Ae. tauschii*, *T. turgidum* ssp. Dicoccoides, and *T. aestivum* genomes encode six, 10, 20, and 30 *wALOGs*, respectively (Table 2). The copy numbers of the *wALOG* family genes were not lost during wheat evolution, suggesting a conserved evolutionary pattern for the wALOG family with wheat polyploidization. In addition, we found that the *wALOG* genes were located on chromosomes one, two, three, six, and seven and were not distributed equally across all these chromosomes, with the number of *wALOG* members being most enriched in the 1A, 1B, 1D, 6A, 6B, and 6D chromosomes (Table 2).

### 3.4. Phylogenetic Analysis and Classification of the wALOG Genes

**Figure 2:**
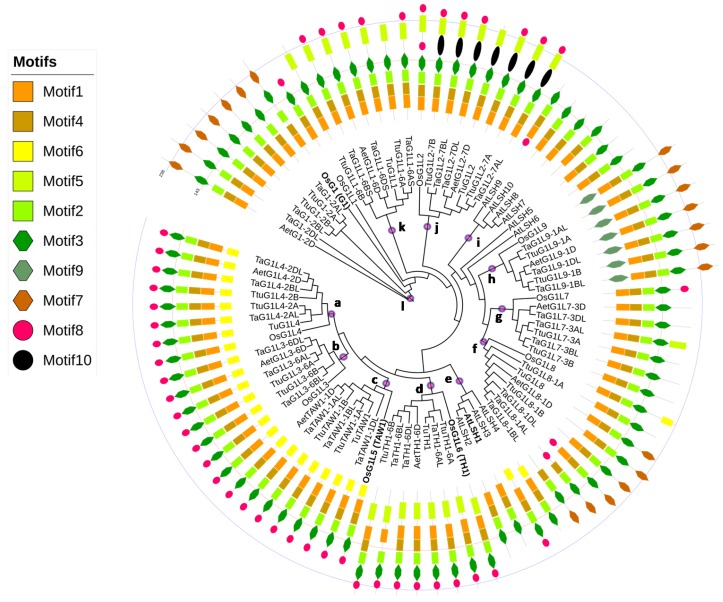
Phylogenetic relationship and conserved motifs of the selected ALOGs. A total of 86 ALOG proteins from *Arabidopsis thaliana, Oryza sativa* subsp. Japonica, *Triticum urartu* (AA), *Aegilops tauschii* (DD), *Triticum turgidum* ssp. Dicoccoides (AABB), and *Triticum aestivum* (AABBDD) were selected to construct the phylogenetic tree and identify the conserved motifs. The blue dots filled with red in the clades and the letters (a–l) show the 12 main groups. The genes written in bold font have known functional *ALOG* genes in *Arabidopsis* and rice. The names of species are abbreviated to two or three letters and detailed information is provided in Table 2. Each conserved motif is illustrated with a specific color and shape, and the distribution of the motifs corresponds to their positions. The first line labels the 143rd amino acid and the second line labels the 238th amino acid position in all sequences.

To determine the evolutionary relationships between the identified wALOGs, the amino acid sequences of the 86 ALOGs from *Arabidopsis thaliana*, *O. sativa subsp. Japonica*, *T. urartu*, *Ae. tauschii*, *T. turgidum* ssp. Dicoccoides, and *T. Aestivum* were used to construct a circular phylogenetic tree using the neighbor-joining method. Based on the topology of the phylogenetic tree, the ALOG proteins were characterized into 12 main groups (a–l), of which groups e and i contained ALOG members derived from Arabidopsis whereas the other groups only contained ALOG members from wheat and rice (Figure 2), suggesting that *ALOG* genes may have evolved independently in eudicots and monocots. In diploid ancestral species (*T. urartu* or *Ae. tauschii*), every wALOG member could be assigned to a separate group. In general, each group had twice as many genes in *T. turgidum* ssp. Dicoccoides and three times as many genes in *T. aestivum* than in *T. urartu*, *Ae. tauschii*, and rice, implying that the copy number of the ALOG family was conserved along with wheat polyploidization. However, the number of ALOGs in each of these groups was lower in *T. urartu* than in the other monocots, which suggested that this may be due to the incomplete *T. urartu* genome. The physicochemical features of the isoelectric points (pIs) and molecular weights (MWs) were calculated using the ProtParam tool [66]. The results revealed that the pIs of these 86 ALOGs ranged from 6.00 to 10.55 with a larger variation in group h, where the MWs of ALOGs varied from 18.52 to 29.94 kDa (Table S2).

To investigate the sequence conservation and divergence between ALOG members belonging to different groups, conserved motifs were identified and illustrated. We found that the motif structure of ALOG proteins was conserved between homologs within each phylogenetic group (Figure 2). Furthermore, motifs 1–4 were present in all ALOG proteins, with the exception of *TaG1L8-1AL*, *TaG1L8-1BL*, and *TaTH1-6BL*. The type of motif present was most conserved among the proteins within groups a–c, and the wALOGs in these groups possessed the distinctive motifs of Motif6. Motif9 was specific to *ALOG* genes in group h and Motif10 was specific to *ALOG* genes in group j, while the other motifs showed an unequal distribution among the groups.

Previous studies have shown that *G1*, *TAW1*, and *TH1* function in mediating the phase transition of IMs [5,18,22]. Our data showed that six *wALOGs* belonged to group l, which shared the highest homology with the *G1* gene of known function, seven *wALOGs* belonged to group d, which shared the highest homology with the *TH1* gene of known function, and seven *wALOGs* belonged to group c, which shared the highest homology with the *TAW1* gene of known function (Figure 2).

### 3.5. Prediction of Cis-Acting Regulatory Elements in the Promoter Regions of wALOGs

In DNA sequences, gene promoters are located upstream of the gene-coding regions and contain multiple cis-acting elements, which are specific binding sites for the proteins involved in the initiation and regulation of transcription [67]. Studies of cis-acting elements are crucial for understanding gene regulation at the transcriptional level. To understand the possible roles of cis-regulatory elements in the regulation of *wALOG* genes in tetraploid wheat, we analyzed the promoter region (comprising 2000 bp upstream of the translation start site) of *wALOGs* in the genome of AABB, which included 20 *TtuALOG* genes and 20 *TaALOG* genes. A total of 116 types of cis-regulatory elements were identified, which could be classified into seven groups (abiotic, biotic, tissue-specific, core, light responsive, circadian, and cell cycle) based on their functions (Table S3). Interestingly, the abiotic group contained 19 members, including 13 (68.42%) involved in the response to hormones such as acetic acid (IAA), gibberellin, abscisic acid (ABA), ethylene, salicylic acid, and methyl jasmonate (MeJA). Light-responsive cis-regulatory elements comprised 29 members (25.00% of the total cis-regulatory elements). These results suggested that hormones and light may play an important role in *wALOG* gene expression.

### 3.6. Expression of the TtALOG Genes and Possibly their Downstream Genes Relate to BM Development

Although some ALOG family proteins share conserved functions, their distinctive expression patterns have led to divergent functions in the regulation of meristem activity and phase transition [29]. *TAW1* has been demonstrated to activate three downstream *SVP* subfamily genes, namely *OsMADS22*, *OsMADS47*, and *OsMADS55*, to promote or prolong the BM identity [5].

**Figure 3:**
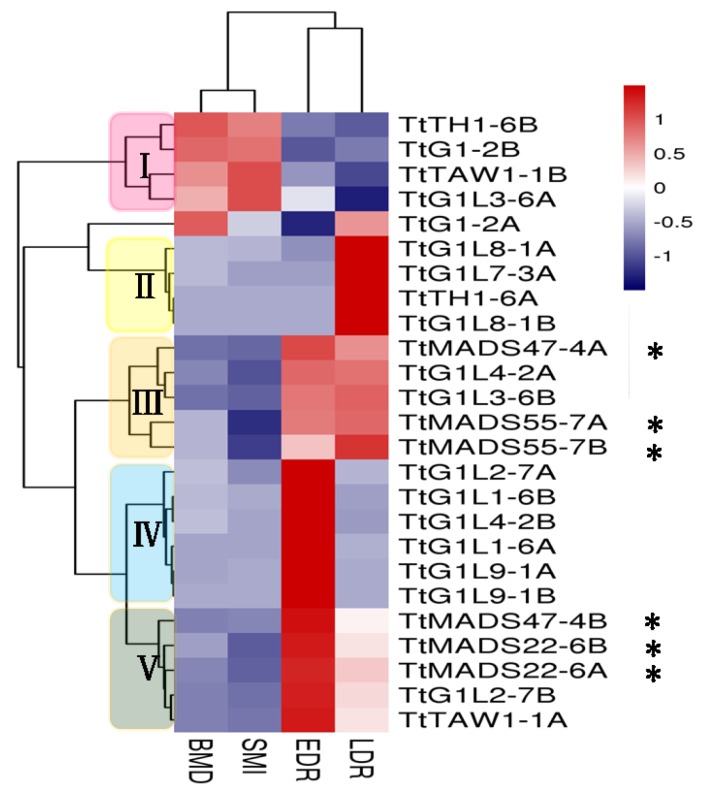
Heat map showing the expression profiling of the *TtALOG*, *TtMADS22*, *TtMADS47*, and *TtMADS55* genes at the early stages of inflorescence development in GAN-A631 (bh). The relative expression levels are shown in the heat map by a gradient of color: blue/white/red (low to high). Abbreviations: EDR, the early double ridge stage; LDR, the late double ridge stage; BMD, the branch meristem development stage; SMI, the spikelet meristem-initiated stage. The heat map color key gradient ranging from global minimum (blue) −1 to global maximum (red) +1 is shown on the right side. Asterisks indicate the *TtMADS22*, *TtMADS47*, and *TtMADS55* of GAN-A631.

To understand the functions of the *TtALOGs* involved in the regulation of BM development in branching head wheat, the dynamic expression patterns of *TtALOG* genes and homologous genes of three *SVP* subfamily genes of rice, *TtMADS22*, *TtMADS47*, and *TtMADS55* of GAN-A631, which displays the branching head phenotype, were investigated with confidence (FPKM > 1) by RNA-seq (Figure 3). The results revealed that all 20 *TtALOG* family genes (with the exception of *TtG1L7-3B*) and six *TtSVP* subfamily genes (*TtMADS22-6A*, *TtMADS22-6B*, *TtMADS47-4A*, *TtMADS47-4B*, *TtMADS55-7A*, and *TtMADS55-7B*) could be classified into five main groups (I–V) according to their expression variation patterns (Figure 3). The members in group I were highly expressed in the BM development and SMI stages, those of group II were only highly expressed in the LDR stage, those of group III were highly expressed in the EDR and LDR stages concurrently and those of group IV were highly expressed in the EDR stage, while those of group V were highly expressed in the EDR stage and relatively weakly expressed in the LDR stage. The expression patterns of most *TtALOG* genes, with the exception of *TtG1L8-1A*, *TtG1L8-1B*, *TtG1L9-1A*, *TtG1L9-1B*, *TtG1L1-6A*, and *TtG1L1-6B*, which are located on chromosome-A, appeared to be divergent from their homologous genes located on chromosome-B, implying the functional diversification of *TtALOGs* after gene duplication. *TtTAW1-1A* (group V) and *TtTAW1-1B* (group I) shared the highest homology with *TAW1*, *TtG1-2A* (an independent group), and *TtG1-2B* (group I) shared the highest homology with *G1*, and *TtTH1-6A* (group II) and *TtTH1-6B* (group I) shared the highest homology with *TH1*. Furthermore, based on the known functions of *TAW1*, *TH1*, and *G1* [5,18,22], the *TtALOG* genes in groups III–V, which were highly expressed in the EDR and LDR stages, may have played a positive role in improving lateral BM development. We also found that all six *TtSVP* subfamily genes belonged to groups III and V, suggesting that these genes may function downstream of *TtALOGs*. It was concluded that the *TtALOGs* of groups III–V were attractive candidate genes for BM development.

**Figure 4:**
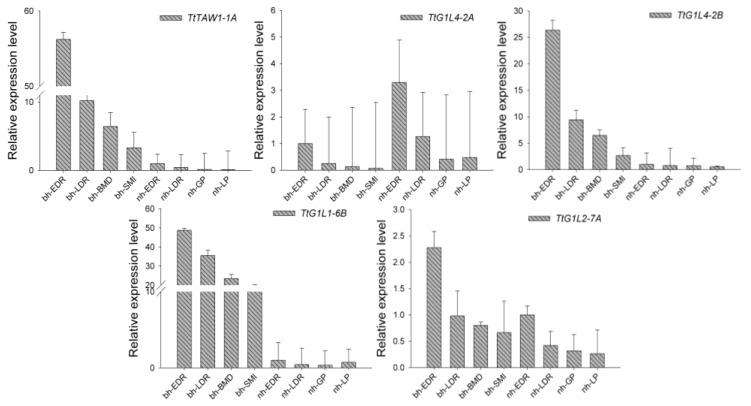
Relative abundance of some ALOG homolog mRNAs as determined by quantitative real-time polymerase chain reaction (qRT-PCR) with samples from the spikes of GAN-A631 (bh) and GAN-A1582 (nh) at the early developmental stages of the spike. The values shown correspond to the mean values for three biological and three technical replicates. Spike developmental stages were assigned as follows, EDR, early double ridge stage; LDR, late double ridge stage; BMD, branch meristem development stage; SMI, spikelet meristem-initiated stage; GP, glume primordia stage; and LP, lemma primordia stage. The use of the bh and nh prefixes means that the samples came from GAN-A631 (bh) and GAN-A1582 (nh), respectively.

To further verify the functions of groups III–V in BM development, the expression patterns of their genes, *TtTAW1-1A*, *TtG1L4-2A*, *TtG1L4-2B*, *TtG1L1-6B*, and *TtG1L2-7A*, were investigated by qRT-PCR analysis in GAN-A631 and GAN-A1582 (Figure 4). The results showed that the expression of these genes was downregulated during the EDR, LDR, BM development, and SMI stages in GAN-A631 as well as during the EDR, LDR, GP, and LP stages in GAN-A1582. (Figure 4). In contrast to normal head wheat, the expression levels of *TtTAW1-1A*, *TtG1L4-2B*, and *TtG1L1-6B* were much higher in branching head wheat, especially for *TtTAW1-1A* (homologous gene of *TAW1*). The mRNA expression of *TtTAW1-1A* and *TtG1L4-2B* was high in tissues with the BM identity relative to other tissues. Furthermore, *TtTAW1-1A* expression was higher than that of the housekeeping genes, whereas all the other genes showed relatively low expression levels, indicating that it may play an important role in the BM identity of branching head wheat.

### 3.7. Characterization of Protein Sequences and Promoters of TtALOG Genes Involved in BM Development

**Figure 5:**
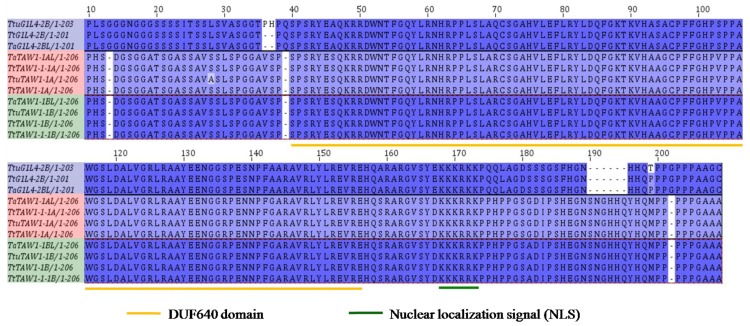
Analysis of the mutant ALOG proteins among wheat. The ALOG proteins were cloned from GAN-A631 (bh) and GAN-A1582 (nh) and their amino acid sequences were compared with those from the *T. aestivum* and *T. turgidum* ssp. Dicoccoides genomes. A conserved region and a nuclear localization signal are indicated with orange and green lines, respectively. GAN-A631 sequences: *TtTAW1-1A* and *TtTAW1-1B*. GAN-A1582 sequences: *TtTAW1-1-1A* and *TtTAW1-1-1B*.

The genes and promoter sequences of the *TtTAW1-1A* (NCBI: MH753535, MH753536, MH753537, and MH753538) and *TtTAW1-1B* (NCBI: MH753539, MH753540, MH753541, and MH753542) loci from *T. turgidum* L. (GAN-A1582 and GAN-A631) were cloned and compared with those of *T. aestivum* and *T. turgidum* ssp. Dicoccoides for the A and B subgenomes (Figure 5 and Figure S1). Compared with the *T. aestivum* protein-coding regions of the A subgenome, a single amino acid substitution was found in *T. turgidum* ssp. Dicoccoides, whereas no sequence differences were found between GAN-A1582 and GAN-A631 (Figure 5). Numerous single-nucleotide polymorphisms (SNPs) were identified in the promoters of the A subgenome loci when comparing *T. aestivum*, *T. turgidum* ssp. Dicoccoides, and *T. turgidum* L., but only one SNP was found between GAN-A1582 and GAN-A631 (Figure S1). Compared with the *T. aestivum* protein-coding regions of the B subgenome, only one consistent single amino acid substitution was found between *T. turgidum* ssp. Dicoccoides and *T. turgidum* L. (Figure 5). A high number of sequence polymorphisms were detected in the promoters of the B subgenome loci among *T. aestivum*, *T. turgidum* ssp. Dicoccoides, and *T. turgidum* L., but only two SNPs were found between GAN-A1582 and GAN-A631 (Figure S1). Finally, low nucleotide diversity was detected between GAN-A1582 and GAN-A631.

The genes and promoters of *TtG1L4-2A* (NCBI: MH753543 and MH753544), *TtG1L4-2B* (NCBI: MH753545 and MH753546), *TtG1L1-6A* (NCBI: MH753547 and MH753548), and *TtG1L1-6B* (NCBI: MH753549 and MH753550) were also cloned from GAN-A631 and compared with those of the *T. aestivum* and *T. turgidum* ssp. Dicoccoides genomes. Compared with the *TtuG1L4-2B* protein sequence, two consistent amino acid substitutions and insertions were found in *TtG1L4-2B* and *TaG1L4-2B*, while the others shared the same protein sequence (Figure 5). Some SNPs, insertions or deletions were detected in the promoters of these gene loci when comparing *T. aestivum*, *T. turgidum* ssp. Dicoccoides, and GAN-A631 (Figure S1).

## 4. Discussion

### 4.1. The Development Pattern of the Branch-Like Meristems of GAN-A631 is Similar to the Indeterminate BMs of Rice

The rice inflorescence has branched structures and is classified as a panicle. In the early stages, the rice IM produces a primary branch, which then produces several lateral meristems [68]. Early lateral meristems acquire an indeterminate BM identity and grow as secondary branches, while later lateral meristems are specified as SMs. The timing of the meristem phase shift from a BM to an SM determines the pattern of branching [4,6]. Normally, the wheat IM does not produce any lateral BMs from the inflorescence main axis and the SMs are initiated during the EDR stage [69]. For example, in the EDR stage of GAN-A1582, SMs emerge to produce spikelets on the main axis of the young inflorescence. However, in the EDR stage of GAN-A631, branch-like meristems emerge on the main inflorescence axis to produce long lateral branches, while later, secondary SMs initiate from the developed branch-like meristems during the SMI stage. Therefore, the development pattern of the branch-like meristems of GAN-A631 was similar to the indeterminate BMs of rice. 

### 4.2. Copy Number Variation and Functional Diversification of wALOGs During Polyploidization

The silencing and elimination of duplicated genes frequently occur during polyploidization, and the retained genes may undergo subfunctionalization or neofunctionalization, leading to functional diversification [70,71]. In previous studies, the copy numbers of seven investigated gene families in hexaploid wheat were considerably lower than the sum of three diploid ancestral species [31,72]. However, the copy numbers were not lost during wheat polyploidization, which means that *wALOGs* may play an important role in the inflorescence development of wheat. Differential expression patterns were observed among *TtALOG* family members and the homologous copies of A and B subgenomes. These results agreed with those of a previous study reporting unequal contributions of the A and B subgenomes towards gene expression [73]. The distinctive expression patterns of *TtALOG* genes indicate that *TtALOGs* play divergent roles in regulating phase transition from branch-like meristems into SMs.

### 4.3. TtALOGs are Attractive Candidates for Promoting Indeterminate BM Fate

Our analysis revealed that *wALOG* family members belong to 10 main phylogenetic groups (a–d, f–h, and j–l) and are evolutionarily conserved between wheat and rice. As a unique regulator in promoting indeterminate BM fate and suppressing the determinate SM fate in rice, *TAW1* shows the highest expression in BMs and disappears when meristems acquire an SM identity [5]. *TtTAW1-1A* and *TtTAW1-1B* shared the highest homology with the *TAW1*, and the expression characteristics of them indicated that the major functional role of *TtTAW1-1A* is in promoting indeterminate BM fate in GAN-A631. Moreover, the low nucleotide diversity of gene sequences and promoters of *TtTAW1-1A* detected by Sanger sequencing between GAN-A1582 and GAN-A631 may suggest that regulating the transition of indeterminate BM fate into determinate SM fate of GAN-A631 is not responsible for the variations in nucleotide sequence observed. *TtG1L4-2A* and *TtG1L4-2B* shared the highest homology with the *OsG1L4* gene of unknown function, and these genes shared similarly distributed motifs and expression patterns with *TtTAW1-1A*, indicating that they may share similar functions. *TtG1L1-6A* and *TtG1L1-6B* shared the highest homology with the *OsG1L1* gene of unknown function, and the expression patterns of *TtG1L1-6B* indicated that it plays an important role in the IM development of GAN-A631. Variation in nucleotides (including some SNPs, insertions, and deletions) in the promoters of *TtG1L4-2A*, *TtG1L4-2B*, *TtG1L1-6A*, and *TtG1L1-6B* was detected among *T. aestivum*, *T. turgidum* ssp. Dicoccoides, and GAN-A631, which provided a reference for further investigations into polymorphic sites in influencing gene transcription. Interestingly, *OsMADS22*, *OsMADS47*, and *OsMADS55* have already been shown to function downstream of *TAW1* [5], and we predict that the *TtALOGs* belonging to groups III–V may work upstream of *TtMADS22*, *TtMADS47*, and *TtMADS55*. Rice *G1* determines sterile lemma identity by suppressing lemma identity, and is highly expressed in glumes [22]. *TtG1-2A* and *TtG1-2B* grouped into a clade within *G1* and showed stable expression patterns before GP initiation, with the lowest expression levels in the EDR stage, suggesting that these genes were good candidates for GP differentiation in wheat. Rice *TH1* suppression of the lateral development of spikelets was highly expressed in young inflorescence, lemma and palea of spikelets [18,74]. *TtTH1-6A* and *TtTH1-6B* grouped into a clade along with the *TH1*; however, *TtTH1-6A* exhibited selectively high expression in the LDR stage of GAN-A631, indicating functional divergence with *TtTH1-6B*.

### 4.4. Light and Phytohormones May Participate in the Regulation of wALOGs

Signals relating to light, water, energy, nutrients, and temperature affecting the determinants of plant inflorescence branching. Phytohormones also play a key role in the control of floral organ morphogenesis. Light and hormone responses depend on the activities of receptors as well as the downstream response elements that mediate complex tissue specific changes [75]. In this study, promoter analysis displayed the presence of light and hormone (including auxin, gibberellin, ABA, ethylene, MeJA, and salicylic acid) response elements regulating the expression of *wALOG* genes (Table S3). This indicated that light and hormones may act as important regulatory factors in the expression of *wALOG* genes. Previous research has indicated that dynamic changes in endogenous plant hormones (auxins, gibberellin, ABA, ethylene, and MeJA) and the differential expression of hormone-related genes were closely associated with the initiation and development of flower organs [76,77,78,79]. Relatively high levels of auxin were beneficial for promoting the formation and elongation of branched rachis in branched spike wheat [76,78,79]. However, it is not clear whether the dynamic changes in these hormones can transform the levels of wheat *ALOG* gene transcription in the regulation of inflorescence branching development. Future work in this area will benefit our understanding of the function of *wALOG* genes.

## 5. Conclusions

In this study, we have identified six, 10, 20, and 30 *wALOG* genes in *T. urartu*, *Ae. tauschii*, *T. turgidum* ssp. Dicoccoides, and *T. aestivum* genomes, respectively. These genes were found to have evolved independently in eudicots and monocots and were evolutionarily conserved between wheat and rice as well as during wheat polyploidization. The prediction of cis-acting regulatory elements in the promoter regions of some of the *wALOG* genes indicated that light and hormones may act as important regulatory factors of *wALOG* gene expression.

Further RNA-seq in branching head wheat and qRT-PCR study in two different wheat genotypes (“normal head” and “branching head”) revealed that *TtTAW1-1A*, a *TAW1*-homologous gene, play a significant role in regulating the transition of indeterminate BM fate into determinate SM fate and some *TtALOG* genes may act upstream of the *TtMADS22*, *TtMADS47*, and *TtMADS55* genes. A set of *TtALOG* genes and promoter sequences and their expression profiles were characterized, and our findings contribute further knowledge on BM development in wheat.

## Figures and Tables

**Table 1:** Summary of transcriptome sequencing data.

Groups	Total Reads	Clean Reads	GC (%)	Q20 (%)	Total Mapped Reads	Ratio (%)
EDR_1	72,894,272	70,725,372	54.97	97.31	64,059,309	90.57%
EDR_2	67,545,684	65,579,864	54.88	96.23	58,973,520	89.93%
EDR_3	51,128,478	50,309,938	52.31	96.19	46,677,546	92.78%
LDR_1	66,259,258	64,664,774	55.30	97.24	58,684,655	90.75%
LDR_2	68,195,516	66,651,562	55.49	96.09	59,891,457	89.86%
LDR_3	47,152,170	46,527,636	52.47	96.45	43,373,007	93.22%
BMD_1	66,756,912	65,234,314	55.94	96.03	58,293,385	89.36%
BMD_2	63,734,100	63,575,894	52.47	98.21	59,187,411	93.10%
BMD_3	44,215,840	42,328,284	54.78	94.24	36,970,389	87.34%
SMI_1	59,302,160	57,670,038	55.70	96.05	51,464,811	89.24%
SMI_2	63,615,448	62,426,934	54.75	96.01	56,088,433	89.85%
SMI_3	48,891,176	48,768,792	52.50	98.21	45,299,165	92.89%

**Table 2:** Homologous copies of the wheat Arabidopsis *LSH1* and Oryza *G1* (ALOG) genes.

No.	Phylogenetic Groups	*Triticum turgidum* ssp. Dicoccoides Genome ID	Gene Name	Gene Name	Ensembl ID	Gene Name	*Triticum urartu* Genome ID	Gene Name	*Aegilops tauschii* Genome ID	Gene Name
		*Triticum turgidum* ssp. Dicoccoides	Corresponding Gene in *Triticum turgidum* ssp. Dicoccoides	Corresponding Gene in *Triticum turgidum* L.	*Triticum aestivum*	Corresponding Gene in *Triticum aestivum*	*Triticum urartu*	Corresponding Gene in *Triticum urartu*	*Aegilops tauschii*	Corresponding gene in *Aegilops tauschii*
1	f	TRIDC1AG044060	*TtuG1L8-1A*	*TtG1L8-1A*	TRIAE_CS42_1AL_TGACv1_002086_AA0038570	*TaG1L8-1AL*	TuG1812G0100003368.01	*TuG1L8*		
2		TRIDC1BG049870	*TtuG1L8-1B*	*TtG1L8-1B*	TRIAE_CS42_1BL_TGACv1_030408_AA0089770	*TaG1L8-1BL*				
3					TRIAE_CS42_1DL_TGACv1_062292_AA0211930	*TaG1L8-1DL*			AET1Gv20705800	*AetG1L8-1D*
4	c	TRIDC1AG023480	*TtuTAW1-1A*	*TtTAW1-1A*	TRIAE_CS42_1AL_TGACv1_000217_AA0006430	*TaTAW1-1AL*	TuG1812G0100001884.01	*TuTAW1*		
5		TRIDC1BG028470	*TtuTAW1-1B*	*TtTAW1-1B*	TRIAE_CS42_1BL_TGACv1_033389_AA0139150	*TaTAW1-1BL*				
6					TRIAE_CS42_1DL_TGACv1_061798_AA0203560	*TaTAW1-1DL*			AET1Gv20402400	*AetTAW1-1D*
7	h	TRIDC1AG033590	*TtuG1L9-1A*	*TtG1L9-1A*	TRIAE_CS42_1AL_TGACv1_002992_AA0046990	*TaG1L9-1AL*				
8		TRIDC1BG038970	*TtuG1L9-1B*	*TtG1L9-1B*	TRIAE_CS42_1BL_TGACv1_031074_AA0107090	*TaG1L9-1BL*				
9					TRIAE_CS42_1DL_TGACv1_061913_AA0205560	*TaG1L9-1DL*			AET1Gv20554400	*AetG1L9-1D*
10	a	TRIDC2AG055200	*TtuG1L4-2A*	*TtG1L4-2A*	TRIAE_CS42_2AL_TGACv1_097378_AA0324030	*TaG1L4-2AL*	TuG1812G0200004320.01	*TuG1L4*		
11		TRIDC2BG057970	*TtuG1L4-2B*	*TtG1L4-2B*	TRIAE_CS42_2BL_TGACv1_129460_AA0384820	*TaG1L4-2BL*				
12					TRIAE_CS42_2DL_TGACv1_159566_AA0539920	*TaG1L4-2DL*			AET2Gv20853200	*AetG1L4-2D*
13	l	TRIDC2AG035710	*TtuG1-2A*	*TtG1-2A*	TRIAE_CS42_2AL_TGACv1_092935_AA0267510	*TaG1-2AL*				
14		TRIDC2BG039660	*TtuG1-2B*	*TtG1-2B*	TRIAE_CS42_2BL_TGACv1_129607_AA0390240	*TaG1-2BL*				
15					TRIAE_CS42_2DL_TGACv1_159706_AA0541770	*TaG1-2DL*			AET2Gv20574300	*AetG1-2D*
16	g	TRIDC3AG050280	*TtuG1L7-3A*	*TtG1L7-3A*	TRIAE_CS42_3AL_TGACv1_193753_AA0619150	*TaG1L7-3AL*				
17		TRIDC3BG056500	*TtuG1L7-3B*	*TtG1L7-3B*	TRIAE_CS42_3B_TGACv1_220926_AA0724140	*TaG1L7-3BL*				
18					TRIAE_CS42_3DL_TGACv1_249281_AA0844060	*TaG1L7-3DL*			AET3Gv20786800	*AetG1L7-3D*
19	k	TRIDC6AG019090	*TtuG1L1-6A*	*TtG1L1-6A*	TRIAE_CS42_6AS_TGACv1_485429_AA1545270	*TaG1L1-6AS*	TuG1812G0600001461.01	*TuG1L1*		
20		TRIDC6BG025090	*TtuG1L1-6B*	*TtG1L1-6B*	TRIAE_CS42_6BS_TGACv1_513152_AA1632150	*TaG1L1-6BS*				
21					TRIAE_CS42_6DS_TGACv1_542463_AA1721150	*TaG1L1-6DS*			AET6Gv20360000	*AetG1L1-6D*
22	b	TRIDC6AG035110	*TtuG1L3-6A*	*TtG1L3-6A*	TRIAE_CS42_6AL_TGACv1_471514_AA1510200	*TaG1L3-6AL*				
23		TRIDC6BG041790	*TtuG1L3-6B*	*TtG1L3-6B*	TRIAE_CS42_6BL_TGACv1_499492_AA1584200	*TaG1L3-6BL*				
24					TRIAE_CS42_U_TGACv1_642735_AA2122410	*TaG1L3-6DL*			AET6Gv20599400	*AetG1L3-6D*
25	d	TRIDC6AG056190	*TtuTH1-6A*	*TtTH1-6A*	TRIAE_CS42_6AL_TGACv1_472779_AA1525890	*TaTH1-6AL*	TuG1812G0600004039.01	*TuTH1*		
26		TRIDC6BG065670	*TtuTH1-6B*	*TtTH1-6B*	TRIAE_CS42_6BL_TGACv1_500779_AA1609580	*TaTH1-6BL*				
27					TRIAE_CS42_6DL_TGACv1_528245_AA1712740	*TaTH1-6DL*			AET6Gv20927400	*AetTH1-6D*
28	j	TRIDC7AG065080	*TtuG1L2-7A*	*TtG1L2-7A*	TRIAE_CS42_7AL_TGACv1_556874_AA1772610	*TaG1L2-7AL*	TuG1812G0700004994.01	*TuG1L2*		
29		TRIDC7BG058610	*TtuG1L2-7B*	*TtG1L2-7B*	TRIAE_CS42_7BL_TGACv1_576798_AA1855280	*TaG1L2-7BL*				
30					TRIAE_CS42_7DL_TGACv1_604927_AA2003130	*TaG1L2-7DL*			AET7Gv21141300	*AetG1L2-7D*

## Supplementary Materials

Figure S1: Cloned promoters of *TtALOG* genes involved in BM development, Table S1: Primers used in this study, Table S2: The primary sequence features of identified ALOG homologs, Table S3: Classification of cis-regulatory elements.
