# Supplementary material for: Genome-Wide Identification and Characterization of wALOG Family Genes Involved in Branch Meristem Development of Branching Head Wheat
Source: Genes (Basel). 2018 Oct 19;9(10):510. doi: 10.3390/genes9100510 (PMC6209938; doi:10.3390/genes9100510)
Supplement: Supplementary file 1 [file genes-09-00510-s001.zip › WenzhiNan-Supplementary-materials/Supplementary-materials-genes.doc]

**Supplementary materials**

# Genome-wide identification and characterization of *wALOG* family genes involved in the branch meristem development of branching head wheat

Wenzhi Nan^1,2^, Shandang Shi^1^, Diddugodage Chamila Jeewani ^1,3^, Li Quan^1^, Xue Shi^1^***** and Zhonghua Wang^1,^*****

*1 State Key Laboratory of Crop Stress Biology for Arid Areas, College of Agronomy, Northwest A&F University, Yangling* 712100*, China*; [nanwenzhi1@163.com](mailto:nanwenzhi1@163.com) (W.N.); [shishandang@nwsuaf.edu.cn](mailto:shishandang@nwsuaf.edu.cn) (S.S.); [jeewanididdugoda@yahoo.com](mailto:jeewanididdugoda@yahoo.com) (D.J.); [lquan@nwafu.edu.cn](mailto:lquan@nwafu.edu.cn) (L.Q.)

*2* College of Life Science, Yulin University, Yulin 719000, China

3 Grain Legumes and Oil Crops Research and Development Centre, Department of Agriculture, Angunakolapelessa, 82220 Sri Lanka

***** Correspondence: [shixue@nwafu.edu.cn](mailto:shixue@nwafu.edu.cn) (X.S.); [zhonghuawang@nwsuaf.edu.cn](mailto:zhonghuawang@nwsuaf.edu.cn) (Z.W.)

Figure S1. Cloned promoters of *TtALOG* genes involved in BM development.

Tables S1. Primers used in this study.

Table S2. The primary sequence features of identified ALOG homologs.

Table S3. Classification of cis-regulatory elements.

Figure S1. Cloned promoters of *TtALOG* genes involved in BM development. GAN-A631 sequences: *TtTAW1-1A* and *TtTAW1-1B*. GAN-A1582 sequences: *TtTAW1-1-1A* and *TtTAW1-1-1B*.

Tables S1. Primers used in this study.

Primers for qRT-PCR

| **Gene Name** | **Primer Sequences** | **Amplicon Length** |
| --- | --- | --- |
| *TtTAW1-1A* | F:5'-CACCAGTACCACCAGATGC-3' | 116bp |
|  | R:5'-GGGAGTACATAGGATACGTAGG-3' |  |
| *TtG1L4-2B* | F:5'-CAGCGGCAGCTTCCACG-3' | 91bp |
|  | R:5'-CTCGTCGTCGTCGCGTCG-3' |  |
| *TtG1L4-2A* | F:5'-GCAGCAGCTTCCACAGCA-3' | 84bp |
|  | R:5'-GTCCTCGTCATCGATCAGCT-3' |  |
| *TtG1L1-6B* | F:5'-CATGCACGCGCACCTGTT-3' | 115bp |
|  | R:5'-CATCACCATCACGATGTCATC-3' |  |
| *TtG1L2-7A* | F:5'-CGCAGCACTACATGCACGC-3' | 112bp |
|  | R:5'-CTGCTCTGGCCATTGCTGTG-3' |  |
| *β-actin* | F:5'-TGTTGTTCTCAGTGGAGGTTCT-3' | 126bp |
|  | R:5'-CTGTATTTCCTTTCAGGTGGTG-3' |  |

Primers for cloning promoters of *TtALOG* genes

| **Gene Name** | **Primer Sequences** |
| --- | --- |
| *TtTAW1-1A* | F:5'-GCCTAGACGTGCAAGTGTCATG-3' |
|  | R:5'-CTGGAGACATGGTGGGAGTACA-3' |
| *TtTAW1-1B* | F:5'-GTCCAATGCAACACAACTACAAC-3' |
|  | R:5'-CATCACAAACAACGCACAGTAG-3' |
| *TtG1L4-2A* | F:5'-CTACGATCTTTCTTTATGAACTAA-3' |
|  | R:5'-GCAACCGGCGGCGGGAG-3' |
| *TtG1L4-2B* | F:5'-TACCCGCAGAAGAATTGAACT-3' |
|  | R:5'-CATGGATCAGCTGCTGAGC-3' |
| *TtG1L1-6A* | F:5'-CAGCACTCAGTTATAGCAATACAGG-3' |
|  | R:5'-CCTGCGTTGTGCACCTTG-3' |
| *TtG1L1-6B* | F:5'-CGACTATTCTTGTATCCTTCTTCC-3' |
|  | R:5'-CATCACCATCACGATGTCATC-3' |

Primers for cloning CDS of *TtALOG* genes

| **Gene Name** | **Primer Sequences** |
| --- | --- |
| *TtTAW1-1A* | F:5'-ATGGACTTGGTACCACAGCCCGACAG-3' |
|  | R:5'-GGGAGTACATAGGATACGTAGG-3' |
| *TtTAW1-1B* | F:5'-ATGGACCTGATACCACAGCCCGACAG-3' |
|  | R:5'-TCACGCCGCGGCGCCGGGAG-3' |
| *TtG1L4-2A* | F:5'-ATGGACCTGTCGGCGAACCCGGAC-3' |
|  | R:5'-TCAGCAACCGGCGGCGGGAG-3' |
| *TtG1L4-2B* | F:5'-CTGCTCAGCAGCTGATCCATG-3' |
|  | R:5'-TCAGCAACCGGCGGCGGGAG-3' |
| *TtG1L1-6A* | F:5'-ATGGACATGTCTGGCGTGAGCGCG-3' |
|  | R:5'-CTAGTTGAACACGGACAGCGG-3' |
| *TtG1L1-6B* | F:5'-ATGGACATGTCTGGCGTGAGCGCG-3' |
|  | R:5'-CTAGTTGAACACGGACAGCGG-3' |

Table S2. The primary sequence features of identified ALOG homologs.

| ID | genes name | Size (aa) | pI | Mw (Da) |
| --- | --- | --- | --- | --- |
| TRIDC6BG065670.2 | *TtuTH1-6B* | 265 | 9.25 | 27602.48 |
| TRIDC6AG056190.3 | *TtuTH1-6A* | 247 | 9.62 | 25545.28 |
| TRIDC6BG041790.2 | *TtuG1L3-6B* | 200 | 9.91 | 20977.46 |
| TRIDC6AG035110.2 | *TtuG1L3-6A* | 202 | 9.91 | 21166.59 |
| TRIDC6BG025090.1 | *TtuG1L1-6B* | 258 | 9.42 | 27620.44 |
| TRIDC6AG019090.2 | *TtuG1L1-6A* | 237 | 9.51 | 25890.4 |
| TRIDC3BG056500.7 | *TtuG1L7-3B* | 212 | 9.48 | 22154.8 |
| TRIDC3AG050280.6 | *TtuG1L7-3A* | 198 | 9.59 | 20979.58 |
| TRIDC2BG039660.1 | *TtuG1-2B* | 257 | 10.16 | 27487.33 |
| TRIDC2AG035710.4 | *TtuG1-2A* | 260 | 10 | 27833.72 |
| TRIDC2BG057970.2 | *TtuG1L4-2B* | 203 | 9.71 | 21722.18 |
| TRIDC2AG055200.6 | *TtuG1L4-2A* | 201 | 9.71 | 21536.99 |
| TRIDC1BG038970.2 | *TtuG1L9-1B* | 283 | 6.97 | 29759.77 |
| TRIDC1AG033590.2 | *TtuG1L9-1A* | 284 | 6.55 | 29940.02 |
| TRIDC1BG028470.2 | *TtuTAW1-1B* | 206 | 9.8 | 22317.93 |
| TRIDC1AG023480.5 | *TtuTAW1-1A* | 206 | 9.8 | 22273.88 |
| TRIDC1BG049870.1 | *TtuG1L8-1B* | 238 | 9.72 | 24899.8 |
| TRIDC1AG044060.2 | *TtuG1L8-1A* | 209 | 9.89 | 22655.85 |
| TRIDC7AG065080.4 | *TtuG1L2-7B* | 268 | 9.53 | 28641.3 |
| TRIDC7BG058610.2 | *TtuG1L2-7A* | 268 | 9.53 | 28570.19 |
| TRIAE_CS42_6DL_TGACv1_528245_AA1712740.1 | *TaTH1-6DL* | 252 | 9.51 | 26172.96 |
| TRIAE_CS42_6BL_TGACv1_500779_AA1609580.1 | *TaTH1-6BL* | 255 | 9.44 | 26334.98 |
| TRIAE_CS42_6AL_TGACv1_472779_AA1525890.1 | *TaTH1-6AL* | 252 | 9.51 | 26202.98 |
| TRIAE_CS42_U_TGACv1_642735_AA2122410.1 | *TaG1L3-6DL* | 202 | 9.91 | 21136.56 |
| TRIAE_CS42_6BL_TGACv1_499492_AA1584200.1 | *TaG1L3-6BL* | 200 | 9.91 | 20977.46 |
| TRIAE_CS42_6AL_TGACv1_471514_AA1510200.1 | *TaG1L3-6AL* | 202 | 9.91 | 21166.59 |
| TRIAE_CS42_6DS_TGACv1_542463_AA1721150.1 | *TaG1L1-6DS* | 259 | 9.42 | 27866.71 |
| TRIAE_CS42_6BS_TGACv1_513152_AA1632150.1 | *TaG1L1-6BS* | 260 | 9.42 | 27881.72 |
| TRIAE_CS42_6AS_TGACv1_485429_AA1545270.1 | *TaG1L1-6AS* | 259 | 9.3 | 27947.78 |
| TRIAE_CS42_3DL_TGACv1_249281_AA0844060.1 | *TaG1L7-3DL* | 212 | 9.51 | 22085.74 |
| TRIAE_CS42_3B_TGACv1_220926_AA0724140.1 | *TaG1L7-3BL* | 212 | 9.48 | 22154.8 |
| TRIAE_CS42_3AL_TGACv1_193753_AA0619150.1 | *TaG1L7-3AL* | 212 | 9.48 | 22146.82 |
| TRIAE_CS42_2DL_TGACv1_159706_AA0541770.1 | *TaG1-2DL* | 269 | 9.84 | 28858.88 |
| TRIAE_CS42_2BL_TGACv1_129607_AA0390240.1 | *TaG1-2BL* | 257 | 10.16 | 27487.33 |
| TRIAE_CS42_2AL_TGACv1_092935_AA0267510.1 | *TaG1-2AL* | 260 | 10 | 27833.72 |
| TRIAE_CS42_2DL_TGACv1_159566_AA0539920.1 | *TaG1L4-2DL* | 201 | 9.71 | 21474.92 |
| TRIAE_CS42_2BL_TGACv1_129460_AA0384820.1 | *TaG1L4-2BL* | 201 | 9.71 | 21514.94 |
| TRIAE_CS42_2AL_TGACv1_097378_AA0324030.1 | *TaG1L4-2AL* | 201 | 9.71 | 21536.99 |
| TRIAE_CS42_1DL_TGACv1_061913_AA0205560.1 | *TaG1L9-1DL* | 284 | 6.55 | 29901.97 |
| TRIAE_CS42_1BL_TGACv1_031074_AA0107090.1 | *TaG1L9-1BL* | 283 | 6.97 | 29759.77 |
| TRIAE_CS42_1AL_TGACv1_002992_AA0046990.1 | *TaG1L9-1AL* | 284 | 6.98 | 29881.98 |
| TRIAE_CS42_1DL_TGACv1_061798_AA0203560.1 | *TaTAW1-1DL* | 206 | 9.8 | 22303.9 |
| TRIAE_CS42_1BL_TGACv1_033389_AA0139150.1 | *TaTAW1-1BL* | 206 | 9.8 | 22303.9 |
| TRIAE_CS42_1AL_TGACv1_000217_AA0006430.1 | *TaTAW1-1AL* | 206 | 9.8 | 22289.88 |
| TRIAE_CS42_1DL_TGACv1_062292_AA0211930.1 | *TaG1L8-1DL* | 233 | 9.72 | 24286.13 |
| TRIAE_CS42_1BL_TGACv1_030408_AA0089770.1 | *TaG1L8-1BL* | 209 | 9.27 | 21168.54 |
| TRIAE_CS42_1AL_TGACv1_002086_AA0038570.1 | *TaG1L8-1AL* | 215 | 9.27 | 21750.37 |
| TRIAE_CS42_7DL_TGACv1_604927_AA2003130.1 | *TaG1L2-7DL* | 268 | 9.64 | 28581.25 |
| TRIAE_CS42_7BL_TGACv1_576798_AA1855280.1 | *TaG1L2-7BL* | 268 | 9.53 | 28618.25 |
| TRIAE_CS42_7AL_TGACv1_556874_AA1772610.1 | *TaG1L2-7AL* | 268 | 9.53 | 28632.29 |
| AET6Gv20927400.2 | *AetTH1-6D* | 252 | 9.51 | 26172.96 |
| AET6Gv20599400.4 | *AetG1L3-6D* | 202 | 9.91 | 21136.56 |
| AET6Gv20360000.1 | *AetG1L1-6D* | 259 | 9.42 | 27866.71 |
| AET3Gv20786800.1 | *AetG1L7-3D* | 212 | 9.51 | 22085.74 |
| AET2Gv20574300.2 | *AetG1-2D* | 266 | 9.83 | 28478.37 |
| AET2Gv20853200.5 | *AetG1L4-2D* | 201 | 9.71 | 21474.92 |
| AET1Gv20554400.1 | *AetG1L9-1D* | 284 | 6.55 | 29901.97 |
| AET1Gv20402400.8 | *AetTAW1-1D* | 208 | 9.8 | 22504.14 |
| AET1Gv20705800.1 | *AetG1L8-1D* | 252 | 10.55 | 26651.1 |
| AET7Gv21141300.4 | *AetG1L2-7D* | 257 | 9.72 | 27547 |
| TuG1812G0100001884 | *TuTAW1* | 206 | 9.8 | 22289.88 |
| TuG1812G0100003368 | *TuG1L8* | 237 | 9.72 | 24662.64 |
| TuG1812G0200004320 | *TuG1L4* | 201 | 9.71 | 21536.99 |
| TuG1812G0600001461 | *TuG1L1* | 259 | 9.3 | 27934.79 |
| TuG1812G0600004039 | *TuTH1* | 252 | 9.51 | 26202.98 |
| TuG1812G0700004994 | *TuG1L2* | 268 | 9.53 | 28627.32 |
| OS05T0347400-01 | *OsG1L9* | 284 | 6 | 29440.37 |
| OS05T0472000-00 | *OsG1L8* | 238 | 9.75 | 24725.63 |
| OS01T0828900-00 | *OsG1L7* | 212 | 9.87 | 21743.25 |
| OS02T0811000-01 | *OsG1L6* | 248 | 9.62 | 25885.66 |
| OS10T0478000-01 | *OsG1L5* | 204 | 9.8 | 21835.46 |
| OS02T0623400-00 | *OsG1L3* | 209 | 9.75 | 21808.32 |
| OS06T0672400-01 | *OsG1L2* | 277 | 9.4 | 29165.48 |
| OS02T0166800-01 | *OsG1L1* | 191 | 10.03 | 21192.11 |
| OS07T0139300-00 | *OsG1* | 276 | 9.45 | 28496.87 |
| OS04T0516200-01 | *OsG1L4* | 202 | 9.86 | 21673.17 |
| AT4G18610.1 | *AtLSH9* | 191 | 9.89 | 21744.52 |
| AT1G16910.1 | *AtLSH8* | 164 | 9.69 | 18524.31 |
| AT1G78815.1 | *AtLSH7* | 195 | 9.86 | 21617.27 |
| AT1G07090.1 | *AtLSH6* | 196 | 9.53 | 21531.32 |
| AT5G58500.1 | *AtLSH5* | 182 | 9.54 | 19689.29 |
| AT3G23290.2 | *AtLSH4* | 195 | 10.2 | 21434.2 |
| AT2G31160.1 | *AtLSH3* | 219 | 9.93 | 24123.1 |
| AT3G04510.1 | *AtLSH2* | 201 | 9.82 | 22774.41 |
| AT2G42610.1 | *AtLSH10* | 177 | 9.55 | 19791.45 |
| AT5G28490.1 | *AtLSH1* | 190 | 10.08 | 21653.5 |
